# Supplementary material for: Maternal interoceptive focus is associated with greater reported engagement in mother-infant stroking and rocking
Source: PLoS One. 2024 Jun 20;19(6):e0302791. doi: 10.1371/journal.pone.0302791 (PMC11189230; doi:10.1371/journal.pone.0302791)
Supplement: S1 File — (DOCX) [file pone.0302791.s002.docx]

**S1 Confirmatory Factor Analysis: Parent-Infant-Caregiving-Touch Scale (Study 1)**
We specified the model according to the original three-factor structure identified by Koukounari et al. (2015), using weighted least squares estimation for our ordinal data which violated multivariate normality. The standardized regression weights are reported in Table 6 and inspection of the estimates showed poor loadings (< .40) for all items on the latent variable measuring Affective Communication (*I kiss my baby* = .36, *I watch my baby* = .17, *I talk to my baby* = .23, *I leave my baby to lie down =* .21)*.* In addition, *I rock my baby,* on the Holding factor, had a loading of .25. The model fitted the data poorly, χ^2^= 2097.852 (51, *N* = 151), p < .001; RMSEA = .108; CFI = .75.

To investigate the model fit, we inspected the modification indices of the items on the original factors. *I rock my baby* loaded more strongly on Stroking and Affective Communication than it did on the Holding subscale, and several items on ​​Affective Communication, such as *cuddle* and *rock*, covaried with the stroking items. This is not surprising, given that these behaviours involve an affective component. We tested a new model, allowing several items to co-vary and removing *I rock my baby* from the Holding subscale and adding it to Affective Communication*.*
**Table 1. Factor loadings for PICT Three Factor Model: Weighted Least Squares (Study 1)**

| **Latent Factor** | **Indicator** | **B** | **SE** | **Z** | **p-value** | **Beta** |
| --- | --- | --- | --- | --- | --- | --- |
| Hold | Hold | 1.000 | 0.000 | NA | NA | **0.925** |
| Hold | Pick up | 1.066 | 0.031 | 33.970 | 0.000 | **0.885** |
| Hold | Cuddle | 0.330 | 0.017 | 19.107 | 0.000 | **0.404** |
| Hold | Rock | 0.715 | 0.036 | 19.884 | 0.000 | 0.253 |
| Stroke | Tummy | 1.000 | 0.000 | NA | NA | **0.801** |
| Stroke | ArmsLegs | 1.020 | 0.012 | 85.376 | 0.000 | **0.828** |
| Stroke | Face | 0.694 | 0.014 | 49.942 | 0.000 | **0.586** |
| Stroke | Back | 0.788 | 0.012 | 63.371 | 0.000 | **0.676** |
| Affective | Kiss | 1.000 | 0.000 | NA | NA | 0.359 |
| Affective | Watch | 0.478 | 0.032 | 14.933 | 0.000 | 0.168 |
| Affective | Talk | 0.293 | 0.025 | 11.964 | 0.000 | 0.227 |
| Affective | Lie down | 1.493 | 0.141 | 10.560 | 0.000 | 0.212 |
| Note: *N* = 151, loadings >.40 in bold | | | | | | |

The second model had an acceptable fit: χ^2^= 984.24 (40, N= 151), p <.001; RMSEA = .082; CFI = .883 (see Table 7 for factor loadings). A Chi-square test of difference found that this model was significantly better than the first model with the initial underlying structure Δ χ2(17) = 1614.8, *p* <.001. However, the loadings for the items on the Holding factor decreased to .60 or less, and the loadings for *I watch my baby, I talk to my baby, I rock my baby* and *I leave my baby to lie down,* on the Affective Communication factor, remained <.40.

**Table 2. Factor loadings for PICT adjusted model: Weighted Least Squares (Study 1)**

| **Latent Factor** | **Indicator** | **B** | **SE** | **Z** | **p-value** | **Beta** |
| --- | --- | --- | --- | --- | --- | --- |
| Hold | Hold | 1.000 | 0.000 | NA | NA | **0.527** |
| Hold | Pick up | 1.273 | 0.057 | 22.52 | 0 | **0.604** |
| Hold | Cuddle | 0.887 | 0.036 | 24.47 | 0 | **0.538** |
| Stroke | Tummy | 1.000 | 0.000 | NA | NA | **0.823** |
| Stroke | Arms legs | 1.033 | 0.011 | 91.16 | 0 | **0.860** |
| Stroke | Face | 0.766 | 0.013 | 58.74 | 0 | **0.629** |
| Stroke | Back | 0.886 | 0.012 | 74.45 | 0 | **0.757** |
| Affective Com | Kiss | 1.000 | 0.000 | NA | NA | **0.507** |
| Affective Com | Watch | 0.724 | 0.033 | 21.94 | 0 | 0.342 |
| Affective Com | Talk | 0.346 | 0.019 | 17.81 | 0 | 0.389 |
| Affective Com | Lie down | 0.374 | 0.090 | 4.15 | 0 | 0.082 |
| Affective Com | Rock | 1.525 | 0.103 | 14.77 | 0 | 0.319 |

*N* = 151, loadings >.40 in bold
