# Supplementary material for: Maternal interoceptive focus is associated with greater reported engagement in mother-infant stroking and rocking
Source: PLoS One. 2024 Jun 20;19(6):e0302791. doi: 10.1371/journal.pone.0302791 (PMC11189230; doi:10.1371/journal.pone.0302791)
Supplement: S2 File — (DOCX) [file pone.0302791.s003.docx]

## **S2 Exploratory analyses with modified Stroking/Rocking factor**

## **Associations between maternal interoceptive focus (MAIA-2) and Stroking/Rocking factor**

When including *I rock my baby* as a fifth item on the Stroking factor, the relationships to the MAIA-2 factors remained statistically significant; *Noticing* (*r* = .17, *p* = .034), *Body Listening* (*r* = .17, *p* = .033) and *Self-Regulation* (*r* = .21, *p* = 012)*.*

## **Predicting Stroking/Rocking from Noticing and Self-Regulation in combined sample of Study 1 and Study 2 (*N* = 262)**

Across the combined sample of Study 1 and 2 (*N* = 262), stepwise multiple regression revealed that a model containing Self-Regulation and Noticing provided the best fit for the data when predicting the modified Stroking/Rocking factor, which included *I rock my baby* in addition to the original stroking items. *Self-Regulation* emerged as the significant predictor and the model explained a statistically significant and weak proportion of variance (3%), *F*(2, 259) = 5.41, *p* = 0.005 (see the model summary below).

|  | **Stepwise regression: Effects of interoceptive focus on engagement in mother-infant stroking/rocking** | | |
| --- | --- | --- | --- |
| *Predictors* | *Estimates* | *CI* | *p* |
| (Intercept) | -0.00 | -0.35 – 0.35 | 1.0000 |
| Self-Regulation | 0.40 | 0.01 – 0.78 | **0.0422** |
| Noticing | 0.31 | -0.07 – 0.69 | 0.1121 |
| Observations | 262 | | |
| R^2^ / R^2^adjusted | 0.040 / 0.033 | | |
