# Supplementary material for: Maternal interoceptive focus is associated with greater reported engagement in mother-infant stroking and rocking
Source: PLoS One. 2024 Jun 20;19(6):e0302791. doi: 10.1371/journal.pone.0302791 (PMC11189230; doi:10.1371/journal.pone.0302791)
Supplement: S3 File — (DOCX) [file pone.0302791.s004.docx]

## **S3 Frequency distribution of body-part words in 10- to 18-month-olds (N = 97)**

The proportion of individual body-part words from the UKI-CDI reported as not understood, understood and/or produced are presented in the following table.

| **Vocabulary item** | **Doesn't know** | **Understands** | **Produces** | **Total understand and produce** |
| --- | --- | --- | --- | --- |
| **Nose** | 42.30% | 41.20% | 16.50% | 57.70% |
| **Hand** | 43.30% | 47.40% | 9.30% | 56.70% |
| **Belly/Tummy** | 45.40% | 46.40% | 8.20% | 54.60% |
| **Feet** | 46.40% | 42.30% | 11.30% | 53.60% |
| **Tooth/Teeth** | 50.50% | 39.20% | 10.30% | 49.50% |
| **Hair** | 51.50% | 35.10% | 13.40% | 48.50% |
| **Head** | 51.50% | 37.10% | 11.30% | 48.40% |
| **Foot** | 56.70% | 36.10% | 7.20% | 43.30% |
| **Mouth** | 60.80% | 34.00% | 5.20% | 39.20% |
| **Ear** | 60.80% | 25.80% | 13.40% | 39.20% |
| **Arm** | 61.90% | 30.90% | 7.20% | 38.10% |
| **Eye** | 61.90% | 24.70% | 13.40% | 38.10% |
| **Face** | 63.90% | 30.90% | 5.20% | 36.10% |
| **Finger** | 67% | 29.90% | 3.10% | 33.00% |
| **Leg** | 70.10% | 24.70% | 5.20% | 29.90% |
| **Toe** | 71.10% | 23.70% | 5.20% | 28.90% |
| **Belly Button** | 73.20% | 20.60% | 6.20% | 26.80% |
| **Tongue** | 75.30% | 22.70% | 2.10% | 24.80% |
| **Back** | 78.40% | 19.60% | 2.10% | 21.70% |
| **Cheek** | 79.40% | 18.60% | 2.10% | 20.70% |
| **Knee** | 80.40% | 14.40% | 5.20% | 19.60% |
| **Chin** | 84.50% | 15.50% | 0% | 15.50% |
| **Lips** | 85.60% | 12.40% | 2.10% | 14.50% |
