# Supplementary material for: Maternal interoceptive focus is associated with greater reported engagement in mother-infant stroking and rocking
Source: PLoS One. 2024 Jun 20;19(6):e0302791. doi: 10.1371/journal.pone.0302791 (PMC11189230; doi:10.1371/journal.pone.0302791)
Supplement: S4 File — (DOCX) [file pone.0302791.s005.docx]

**S4 Confirmatory Factor Analysis: Parent-Infant-Caregiving-Touch Scale (Study 2)**
 The standardized regression weights are reported in Table S5. Inspection of the estimates revealed several poor loadings (*I leave my baby to lie down =* .25, *I kiss my baby =* .18), although, *I watch my baby* and *I talk to my baby* had high loadings (.91 and .82, respectively), unlike in Study 1*.* This suggests the Affective Communication factor is unreliable. The model did not fit the data well: χ^2^= 153.778 (51, *N* = 111), p < .001; RMSEA = .135; CFI = .84.

**Table 1. Factor loadings for PICT Three Factor Model in Study 2: Weighted Least Squares**

| **Latent Factor** | **Indicator** | **B** | **SE** | **Z** | **p-value** | **Beta** |
| --- | --- | --- | --- | --- | --- | --- |
| **Hold** | Hold | 1.000 | 0.000 | NA | NA | **0.881** |
| **Hold** | Pick up | 1.039 | 0.037 | 28.075 | 0.000 | **1.016** |
| **Hold** | Cuddle | 0.885 | 0.055 | 16.159 | 0.000 | **0.872** |
| **Hold** | Rock | 1.035 | 0.116 | 8.913 | 0.000 | **0.435** |
| **Stroke** | Tummy | 1.000 | 0.000 | NA | NA | **0.864** |
| **Stroke** | Arms legs | 0.770 | 0.058 | 13.362 | 0.000 | **0.773** |
| **Stroke** | Face | 0.666 | 0.048 | 13.854 | 0.000 | **0.860** |
| **Stroke** | Back | 0.545 | 0.058 | 9.374 | 0.000 | **0.568** |
| **Affective Com** | Kiss | 1.000 | 0.000 | NA | NA | 0.181 |
| **Affective Com** | Watch | 5.171 | 1.556 | 3.323 | 0.001 | **0.916** |
| **Affective Com** | Talk | 5.128 | 1.505 | 3.408 | 0.001 | **0.823** |
| **Affective Com** | Lie down | 3.363 | 1.178 | 2.855 | 0.004 | 0.255 |

Note: *N* = 151, loadings >.40 in bold

Modification indices suggested that, as in Study 1, *I rock my baby* loaded more strongly onto the Stroking factor. Again, several items on ​​Affective Communication, such as *I watch my baby*, covaried with the stroking items. We tested a new model, which allowed items to covary, and replaced *I rock my baby* from the Holding subscale to Stroking.

Item loadings for the second model are presented in Table S6. After moving *I rock my baby* from the Holding factor to Stroking, its loading improved from .43 to .57. There was improvement of the overall fit with the second model: χ^2^= 80.06 (46, N= 111), p <.01; RMSEA = .08; CFI = .95. A Chi-square test of difference found that this model was significantly better than the first model with the initial underlying structure Δ χ2(5, 111) = 73.72, *p* <.001. Allowing several items to covary, however, meant that across the Affective Communication factor, all the item loadings reduced, and the loadings for *I kiss my baby* and *I leave my baby to lie down,* remained <.40.

**Table 2.** Factor loadings for PICT adjusted model in Study 2: Weighted Least Squares

| **Latent Factor** | **Indicator** | **B** | **SE** | **Z** | **p-value** | **Beta** |
| --- | --- | --- | --- | --- | --- | --- |
| **Hold** | Hold | 1.000 | 0.000 | NA | NA | **0.810** |
| **Hold** | Pick up | 0.974 | 0.048 | 20.148 | 0 | **0.932** |
| **Hold** | Cuddle | 0.970 | 0.073 | 13.346 | 0 | **0.901** |
| **Stroke** | Tummy | 1.000 | 0.000 | NA | NA | **0.889** |
| **Stroke** | Arms legs | 0.763 | 0.052 | 14.565 | 0 | **0.793** |
| **Stroke** | Face | 0.673 | 0.049 | 13.633 | 0 | **0.784** |
| **Stroke** | Back | 0.680 | 0.052 | 13.134 | 0 | **0.719** |
| **Stroke** | Rock | 0.647 | 0.078 | 8.259 | 0 | **0.573** |
| **Affective Com** | Kiss | 1.000 | 0.000 | NA | NA | 0.346 |
| **Affective Com** | Watch | 1.557 | 0.268 | 5.806 | 0 | **0.563** |
| **Affective Com** | Talk | 1.819 | 0.328 | 5.547 | 0 | **0.607** |
| **Affective Com** | Lie down | 1.423 | 0.380 | 3.740 | 0 | 0.209 |

## 
