# Supplementary material for: Maternal interoceptive focus is associated with greater reported engagement in mother-infant stroking and rocking
Source: PLoS One. 2024 Jun 20;19(6):e0302791. doi: 10.1371/journal.pone.0302791 (PMC11189230; doi:10.1371/journal.pone.0302791)
Supplement: S5 File — (DOCX) [file pone.0302791.s006.docx]

## **S5 Correlation checks between interoceptive accuracy and heart rate variables Study 2**

An inverse relationship was found between heart rate and interoceptive accuracy (*r_s_* = -.26, *p* = .05), which is often reported (Ainley et al., 2020). Participants’ estimate of their resting heart rate did not correlate with interoceptive accuracy (*r_s_* = -.03, *p* = .83), suggesting that people’s beliefs about their heart rate did not influence interoceptive accuracy.
