# Supplementary material for: Maternal interoceptive focus is associated with greater reported engagement in mother-infant stroking and rocking
Source: PLoS One. 2024 Jun 20;19(6):e0302791. doi: 10.1371/journal.pone.0302791 (PMC11189230; doi:10.1371/journal.pone.0302791)
Supplement: Attachment — Submitted filename: Rebuttal letter.pdf [file pone.0302791.s007.pdf]

ROYAL HOLLOWAY UNIVERSITY OF LONDON

Royal Holloway
University of London
Egham, Surrey
TW20 0EX

17th July 2023

Dear Editor(s),
This letter outlines why the initial submission was rejected and clarifies the reasons why we believe this decision as wrong .

On Thursday 1st June we received an email from Dr Jayati Bhattacharyya (copied below in **bold fonts**), stating that our submission was rejected due to it not meeting ethical requirements. Dr Jayati Bhattacharyya stated that the reason for this was that we 'obtained ethical approval from your local ethics committee after you began the study', but this was **not** the case. Despite attaching our ethics approval letter from the ethics team to the original submission, clearly stating that ethics was completed for the first study in May 2020 and again for the 2nd study in 2021, the manuscript was rejected.

The first study did not have an ethical approval code. There was a code for the 2nd study, which was dated 1st April 2021. It seems the confusion was because the ethics code was thought to be applied to both studies, hence why it appears that ethics was obtained after the first study. However, as stated in the ethics letter, ethics was obtained for the first study on 5th May 2020.

This was rectified in the manuscript (tracked changes copy shows that I have clarified that the ethics code is only relevant for the 2nd study) and explained in the resubmission.

There were no 'revisions' provided with the rejection email (see the email below), as the rejection was based on an administrative issue and had not been sent to peer review. As aforementioned, this confusion has been explained in this letter, and a clarification of the ethics code has been made in the resubmission. There were no other changes to the manuscript.

We have written more than three times to Dr Bhattacharyya about this issue but we didn't receive any reply. Hence, we decided to resubmit our manuscript, which as we explain above meets the ethics requirements of PLOS ONE.

Sincerely,
Rosie Drysdale, Jeanne Shinskey & Manos Tsakiris

Rosie Drysdale, BSc
Doctoral Candidate,
Royal Holloway University of London
Rosie.drysdale@rhul.ac.uk

Thursday 1st June 2023 18:32
PONE-D-23-12503
Maternal interoceptive focus is associated with greater reported engagement in mother-infant stroking and rocking
PLOS ONE

Dear Dr. Drysdale,

Thank you very much for submitting your manuscript to PLOS ONE, and for responding to our recent requests regarding your submission. After careful evaluation, we have decided that your submission does not meet our publication criteria and must be rejected.

Based on our evaluation of your manuscript and the information you provided, we do not feel that your submission meets our ethical requirements for human subjects research submissions. PLOS ONE requires that research meets all applicable standards for the ethics of experimentation and research integrity (http://journals.plos.org/plosone/s/human-subjects-research). We reserve the right to reject any submission that does not meet our internal ethical standards, which in some cases are more stringent than local ethical standards.

We recognize that you obtained ethical approval from your local ethics committee after you began the study: your ethics approval document is dated 1st April 2021, while participants were recruited beginning in May 2020. For human subjects research, PLOS ONE requires that authors obtain ethics committee approval before beginning the study, and we do not accept retrospective ethical approval.

As a result of these concerns we cannot consider the manuscript for publication at PLOS ONE. I am sorry that we cannot be more positive on this occasion, but hope that you understand the reasons for this decision.

Kind regards,
Jayati Bhattacharyya, PhD
Editorial Office
PLOS ONE
