# Supplementary material for: Maternal interoceptive focus is associated with greater reported engagement in mother-infant stroking and rocking
Source: PLoS One. 2024 Jun 20;19(6):e0302791. doi: 10.1371/journal.pone.0302791 (PMC11189230; doi:10.1371/journal.pone.0302791)
Supplement: Attachment — Submitted filename: Response to reviewers.docx [file pone.0302791.s008.docx]

**Response to reviewers: Revision of PONE-D-23-19284**

Maternal interoceptive focus is associated with greater reported engagement in mother-infant stroking and rocking

PLOS ONE

Academic Editor:
Thank you for submitting your manuscript to PLOS ONE. Please accept our apologies for the length of the review process; this was because it was extremely difficult to find reviewers to assess it. The reviews are below - these are positive but there are some revisions required. Therefore, we invite you to submit a revised version of the manuscript that addresses the points raised during the review process.

***REPLY: Thank you for handling our manuscript. Below we provide a point-by-point reply to all the comments from the reviewers, and we hope that these have addressed all the issues. Our revisions to the manuscript are in red font (in “Revised with track changes.docx”).***

Review Comments to the Author:

Reviewer #1: Many thanks for the opportunity to review this interesting paper. The report demonstrates the important role that interoception could play in parent-infant caregiving behaviours, and outlines the start of the process of understanding the role of interoception in mother-infant dyadic communication, whilst indicating the flaws of particularly the more objective measures of interoceptive accuracy.

It is clear that the research has been conducted thoroughly, and includes thorough analysis of data and conclusions that largely support the results outlined. I would recommend some adjustments to the manuscript, which I feel could further develop the article.

***REPLY: Thank you for reviewing our manuscript. We hope that the replies provided below and the changes in the manuscript address all your comments.***

Major points:
1. Throughout the paper there are issues with the narrative and flow, which make it difficult to follow. There should be a flow throughout of the key hypotheses, from background, to hypotheses, to results then to discussion – some of these are missed at times. This would make the paper easier to follow throughout. Specific examples include:

a. I felt that the explanation of interoception, in relation to the mother-infant relationship was clearly outlined in the introduction, but the narrative relating to body satisfaction and to infant vocabulary was sparce and unclear. For example, an outline or discussion of the competition of cues hypothesis or other explanation why the relationship between exteroception and interoception occurs, would be useful to add context and clarify the relevance of body satisfaction here. This would allow a more holistic explanation of how these concepts fit together.

***REPLY: Thank you for this suggestion, we have (after defining body image) alluded to the relevant literature suggesting the inverse relation between external and internal representation of the body (e.g., Ainley et al., 2012; Emmanuelsen et al., 2015; Eshkevari et al). We have also made sure to explicitly reason as to why this may be relevant to pregnancy/postpartum and with links to caregiving. We now write on page 6, starting from line 128:***
*Moreover, evidence suggests that interoception plays a key role in shaping body image (61–63), defined as the conscious, mostly visual, mental representation of an individual’s body appearance, including its perceptual aspects (e.g., its shape, size) and its affective-cognitive evaluation (e.g., feeling dis/satisfied with the appearance or size of the body) (64). For individuals with have poorer interoceptive abilities, their body representation seems to be more heavily influences by exteroceptive (i.e., visual) features of their body, which may also explain why they are more susceptible to body-image disturbances (65). Accordingly, lower interoceptive accuracy is associated with greater body dissatisfaction (66). The substantial bodily changes experienced during pregnancy and the postpartum suggest these may be sensitive periods for interoception and body image, but few studies have investigated this. In a sample of pregnant women, lower self-reported interoceptive focus was associated with greater body dissatisfaction during pregnancy (67), which has been linked to less engagement in breastfeeding (68), highlighting that interoception and body image have the potential to influence parent-infant caregiving.*

Likewise, there was no mention in the introduction as to why infant vocabulary, and particularly in relation to body parts, might relate to the other variables, yet this was hypothesised. A full explanation of all elements of the hypotheses would have allowed a clearer narrative and flow through the introduction/hypotheses. It may be intuitive to some readers, but explicitly justifying would allow for a clearer narrative in the introduction.

***REPLY: We have included a rationale in the introduction relating caregiving to body part comprehension. We have cited a relevant paper highlighting a role for parents’ verbal communication of interoceptive states (i.e., MacCormack et al, 2020) which fits in with the flow of the section, because it touches on how parental interoception may support infants’ awareness of bodily states through caregiving. We now write, starting from line 107:***

*In childhood, evidence suggests that the way parents verbally communicate their interoceptive states may shape how children identify and appraise their own bodily sensations (52). Given that the words for body parts are among the first words infants learn (53), the caregiving environment may shape children’s identification and labelling of their body and its parts even earlier.*

b. From line 334: The subheading is framed as relating to stroking, but the content of that subsection relates to interoception. It therefore feels like a section of the results is missing – relationships between PICT and other variables.

***REPLY: We have swapped the wording to make this subheading more accurate. The subheading is now “Associations between maternal self-reported interoception and mother-infant stroking, pregnancy body satisfaction and social touch attitudes”. We also explicitly stated the non-significant result between stroking (PICT) and retrospective pregnancy body satisfaction (BUMPS) in that section (which also links to your point d. below).***

c. From line 600: The results for study 2=two are in a different order to those from study one. It would be easier to follow if either the results were in the same order as the hypotheses, or by order of type of analysis (correlations then regressions).

***REPLY: Thanks to the Reviewer’s comments we have now improved the presentation of our results and statistical analysis in Study 2, to match those of Study 1; integrating the correlations together before the regressions as suggested (which links to point d. below). We agree that this is better for consistency and cross referencing between the results of the studies. Thank you for this suggestion.***

d.The results lack acknowledgement of non- significant results. It would be useful to either acknowledge this, or include null results, in the interest of open reporting of data, and to ensure a flowing argument through results and discussion. For example the statement on line 412 “Conversely, engagement in these caregiving behaviours was not associated with pregnancy body satisfaction, nor social touch attitudes.” is outlined as a key outcome in the discussion, but was not outlined in statements in the results – it appeared in the table, but as it is framed as a key result in the discussion it might be useful to overtly outline it in the results section. This detracts from the ability to follow the argument through.

***REPLY: We have explicitly stated the non-significant results i.e., between the subscales of the maia and retrospective body satisfaction at final trimester and postpartum, in the section with the correlations (before the regression). We have also added the contrast between current vs postnatal in the mixed model (as per your point 14 below).***

**Minor points:**

Introduction:

1. Line 65-66: It is unclear how people’s interoceptive insights influence how they interact with users. It might help if this could be made more explicit
***REPLY: Yes we see how this was unclear and sparse. We have cleared this up by removing that phrasing and adding citations to highlight its link to emotion regulation, in the following paragraph (Dedentado et al., 2023; Zamariola et al., 2019). We now write on page 3, starting line 68:***

*Poorer self-reported interoceptive abilities (as reported in the MAIA-2) have been associated with deficits in emotion regulation in healthy populations (20) which contributed to depressive symptomatology (21).*

2. Line 79: Further examples of physiological reactions could further the readers understanding of the impact of infant distress, for example milk let down.

***REPLY: Thank you for this suggestion, we have added the example of stimulating milk flow from the breast, citing Bornstein et al (2017).***

3. Line 124: Your measures are of body (dis)satisfaction, but the introduction refers to the concept of body image. There are subtle differences between these concepts which I feel could be clarified and discussed, possibly with key definitions and distinctions of both, or just referring to body satisfaction throughout.

***REPLY: To resolve this, we have added a brief definition of body image and its relation to body satisfaction (line 124), and more explicitly stated when findings of previous research measured body satisfaction (i.e., rather than using body image as a broad term). For instance, on lines 749 and 750 we have reworded ‘body image concerns’ to ‘body dissatisfaction’ and explicitly outlined what quantified this in that study’s measure e.g., stretch marks, appearance and weight gain. We agree that this should provide more clarity for the reader.***

Method:
4. Line 159: The explanation of the power analysis was clear, but the justification for using a small effect size is missing. Likewise, the process of the power analysis for study 2 is not clear? Presumably if this was the same as for study one, the sample size fell short of this, in which case an explanation/acknowlegement for this would be helpful.

***REPLY: Yes, we attempted to recruit the same number of participants as Study 1. We have acknowledged that our sample size fell short due to difficulties in recruiting because of the stricter inclusion criteria (i.e., participants needed to complete the study on a laptop with a webcam).***

5. Line 172: Incomplete responses were mentioned but it is not clear if the research team excluded the participants for single data points, or just for whole measures that were missing. The paper would benefit from this being clarified.

***REPLY: We have clarified that these were removed because they did not finish the online survey and therefore their responses were not included (lines 178-179).***

6. Line 247: BUMPS is a 19 item measure, but here it is described as 20 items.

***REPLY: Thank you for correcting us on this, we have rectified this in the paper (when referring to it as a 19-item questionnaire and the possible range of scores in the descriptive statistics). We also noticed in the process that we had labelled the max scores in the reporting of the interoception subscales in the descriptives, rather than the means, so we have rectified this too.***

Further to this, it is unclear whether participants were asked about a specific time point in pregnancy when they were recalling retrospectively - Could be clarified, and an acknowledgement included that body (dis)satisfaction changes during pregnancy.

***REPLY: Thank you for the suggestion, in lines 254-259 we have clarified this by explicitly stating that in the original study women responded across all stages of pregnancy (i.e., first through to third trimester), and that in our study we did not ask about a specific time during pregnancy. Later (lines 767-770) we have acknowledged that, as you describe, body (dis)satisfaction changes during pregnancy and this coarse measure of an ‘overall’ pregnancy body satisfaction would not have captured these changes. To the latter point, we now write, starting page 37 (“Interoception and body-image” section):***

*It is worth noting that as body (dis)satisfaction likely changes throughout pregnancy, using the BUMPS to measure (retrospective) accounts of pregnancy body satisfaction overall i.e., not specifying a specific trimester or period, was limiting.*

7. Line 439: The sample appears to be varied, across 3 countries. It would be useful to acknowledge a quick explanation of why those countries were chosen, to possibly pick this up in discussion, in terms of the potential drawbacks of this, e.g., maternity leave differences in UK/USA might cause behavioural differences from mother towards infant.

**REPLY:**

***Thank you for raising this point. For Study 2, we had more inclusion criteria (e.g. “female participants between 18 and 40 years old, with a child born in the year 2020, and with access to a computer or laptop, and consent to be video recorded. There were too few eligible participants on Prolific who met these criteria and we therefore opened up recruitment to other countries with native English speakers, and raising the number of eligible participants to n=380. In that sense, the recruitment from these countries was not theoretically motivated. We ended up with n= 75 from UK, n=2 from Ireland, and n=34 from USA.*** ***While there are differences in maternity leave entitlements across these countries and these may have affected, as the Reviewer suggests, interaction between mothers and infants in the first few years of life, we do not have data on whether and how our participants made use of their maternity leave entitlements. The potential across-countries differences on maternity leave and other factors could play a role on mother-infant interactions, as you suggest, but we do not have any data to speak to that point, however we agree that this is an important research question for future studies that can focus on cross-cultural differences in interoceptive development.***

Results:

8. Table 3 and table 8: Could you clarify what the last column refers to – could it be described as ‘possible range’ rather than ‘range’?

***REPLY: Yes, this is the possible range and we have reworded this accordingly.***

9. Line 370 and 456: It would be useful to explain why those particular subscales of the MAIA have been chosen, as it is unclear in the manuscript.

***REPLY: We have explicitly stated our selection of the MAIA subscales of interest from study 1.***

10. Line 482-483: The paragraph starts: “Before the task, participants had to successfully answer questions regarding these requirements to proceed to the experiment, e.g., “I can complete the study on a mobile”, however the requirements referred to here have not been explained prior to this, but it is written as if it is referring to the previous paragraph, which it is not.
***REPLY: Thank you for spotting this. We have resolved this in relation to your next comments (point 11 and 12) by rewording this section and providing more detail. We hope this is clearer.***

11. Line 494: Sentence “Heart rate estimation was conducted offline using the video recordings” is not clear – can you clarify what is this referring to? Is it what the participants did, or some calculation conducted by the researcher?

***REPLY: Thank you for your suggestion, we initially removed some of the details for brevity but having re-read it we agree that it requires further explanation. We have added further details about the heart rate estimation procedure (and the task as requested below). We have tried to keep this as concise as possible while providing the necessary details to assist the reader's understanding. We now write, starting on page 24 (“Online heartbeat counting task: Remote photoplethysmography” section):***

*Participants completed a measure of interoceptive accuracy that could be obtained remotely. Specifically, the Heartbeat Counting Task (HCT) (13) was integrated with a novel method for calculating heart rate from pixel changes in video recordings of human skin (84). Remote photoplethysmography (rPPG) uses a light source to measure the variation in oxygenated blood flow and corresponds to phasic changes in heart rate (85). rPPG works via photo-amplification, which detects variations in the reflected colours of the skin caused by changes in capillary tissue movement that can be extracted from luminance values in the video recording (86). An algorithm applies signal detection procedures based a step-by-step approach (84), most recently used in (87,88). First, the algorithm automatically detects the presence of a face and extracts facial features, filtering out other irrelevant ones, i.e. hair, teeth, clothing, background. For every video frame and RGB colour channel, the average of all selected pixels was computed and aggregated across time. A filter (0.04Hz) is applied to the raw signal to remove movement artefacts and low-frequency changes in the signal. The most prominent component signal in all colour channels is extracted and converted to a power spectra estimation using fast-Fourier transformation. A high peak in power at a given frequency reflects the heart rate. Low-pass frequency filter was applied to the power spectra (0.02Hz) and the heart rate at the highest power peak across components was selected as the final rPPG heart rate. In a validation study using the rPPG method presented in the current study, Di Lernia (88) showed that heart rate estimates from webcam video recordings converged well with heart rate estimates extracted from a validated mobile application.*

*…* *Heart rate estimation was conducted offline using the video recordings (using the algorithmic approach outlined above). Because HR estimation requires video recordings from a webcam and enough luminance values, participants were instructed to sit still in a room with natural light covering their face. Before the task, participants had to successfully answer questions regarding requirements to proceed to the experiment, e.g., “I can complete the study on a mobile phone” (correct answer: no), “I can perform the experiment at night in a dark room” (correct answer: no) and so forth. The webcam was activated between each experimental trial so that participants could adjust themselves accordingly and they were given one practice trial at the beginning.*

12. Line 496: Can more explanation be given about the processes relating to the interoceptive accuracy task, the instructions etc. This could be followed up in the results with a descriptive summary of their estimations.

***REPLY: In resolving the above point/s, we have added more explanation about the interoceptive accuracy task (in the same (“Online heartbeat counting task: Remote photoplethysmography” section right after the text above). Thank you for this recommendation (our responses to this point and the previous points 10 and 11 can be found in lines 484 - 518).***
*Adopting the rPPG allowed a measurement of heart rate that is required for the measure of interoceptive (cardiac) accuracy in the HCT. Specifically, during the HCT, participants are instructed to focus and count their heartbeats during several short intervals (e.g. 25 s, 35 s, 45 s), without taking their pulse and without guessing. To quantify interoceptive accuracy in the HCT, participants’ reported heartbeats are compared against their actual heartbeats (in this case extracted via the rPPG).*

13. Line 533-539: It would be useful to clarify which time point the alpha for BISS came from, or to give one for each timepoint?

***REPLY: We have clarified that the states alpha for the BISS was that value (i.e., .95) for all time points.***

14. Line 638: Was there an analysis comparing current vs postnatal to add in here, so that there are comparisons of all 3 timeframes?

***REPLY: Thank you, yes, we have added the contrast between current vs. postnatal.***

15. Line 718: Attention regulation (construct captured by the subscale from the MAIA) has been explained, but it would be useful to explain self-regulation too so that the subtle differences between them are clearer.

***REPLY: We had defined the self-regulation scale in the previous lines but for clarity we resolved this by redefining it when we compare it against attention regulation (as you suggested). Accordingly, we added the following on page 36, starting line 865:***

*As previously mentioned, Self-Regulation describes the ability to regulate distress by sustaining attention to body sensations.*

Discussion:
16. Limitations: It would be helpful to acknowledge the small sample size and large proportion of exclusions in study 2 here. Also, the issues of relying on maternal reporting of mother-infant communication – the skewness was mentioned earlier in the report, but not addressed here. Possibly therefore suggesting watching videos or live-video conferencing of dyadic interactions between mother and infant.
***REPLY: We have extended our pre-existing statement about the limitation of the strict exclusion criteria, to explicitly reference the subsequent small sample size. We have also specified recording parent-infant interactions as a future direction. We now write, starting page***

Reviewer #2: This is an interesting paper on an important topic; and I applaud the authors efforts to conduct this type of much needed research.
I have two minor comments:

***REPLY: Thank you for viewing our manuscript. We hope that the replies provided below and the changes in the manuscript address your comments.***

a) the Discussion section (the general) tends to repeat the Discussion 1 and 2. I suggest to present the studies one after the other, and put all the discussion at the end. I find many § repetitive

***REPLY: Thank you, we agree there was some unnecessary repetition. To resolve this, we have now substantially shortened the “discussion of Study 1” section to only include a few sentences behind the theoretical motivation for Study 2 in light of the findings of Study 1, in order to save the critical analysis and discussion for the general discussion section. We now write (under “Discussion of Study 1” section):***

*The key findings of Study 1 was that mothers who reported greater tendencies to focus on, and regulate, their own interoceptive states reported engaging in more stroking and rocking. Conversely, engagement in these caregiving behaviours was not associated with pregnancy body satisfaction, nor social touch attitudes. Aversion to social touch correlated with unique aspects of interoception, i.e., negative styles of distraction and trust towards bodily states. Importantly, these were not relevant to stroking, potentially explaining the lack of relationship. We discuss the interpretation of these results in greater detail in the General Discussion.*

*We designed and implemented Study 2 to extend the potential role of interoception by investigating whether objective interoceptive accuracy also plays a role in maternal engagement in stroking and rocking. We also aimed to replicate our previous finding that mothers who show greater self-reported interoceptive focus also report greater engagement in mother-infant stroking and rocking.*

b) I wonder how much of the patterns you found are related precisely to the fact that the study was conducted during a stressful period for everyone. I believe this needs to be clearly discussed in the paper, not just briefly mentioned in the limitations.

**REPLY: Thank you for your suggestion, we have expanded on this and alluded to its potential effect/s on the sample and the results. However, we also highlight that scores on the MAIA are consistent with previous studies. We now write:**

*Relatedly, it is important to consider that the data were collected during a time when direct and indirect effects (e.g., lockdowns, school closures) on parents’ psychological well-being (112) were particularly heightened for mothers, who were more likely to juggle work and care responsibilities (113). The relation between parents’ own interoceptive abilities i.e., self-regulation, and their caregiving engagement may have been different if examined outside of this context. However, it is also worth noting that the MAIA-2 scores we obtained from our sample are comparable to those of previous studies (14).*

**ADDITIONAL INFO (MOVE TO TOP ONCE DONE):**
Please submit your revised manuscript by Apr 11 2024 11:59PM. If you will need more time than this to complete your revisions, please reply to this message or contact the journal office at plosone@plos.org. When you're ready to submit your revision, log on to <https://www.editorialmanager.com/pone/> and select the 'Submissions Needing Revision' folder to locate your manuscript file.

Please include the following items when submitting your revised manuscript:

- A rebuttal letter that responds to each point raised by the academic editor and reviewer(s). You should upload this letter as a separate file labeled 'Response to Reviewers'.

**REPLY: Included**

- A marked-up copy of your manuscript that highlights changes made to the original version. You should upload this as a separate file labeled 'Revised Manuscript with Track Changes'.

**REPLY: Included**

- An unmarked version of your revised paper without tracked changes. You should upload this as a separate file labeled 'Manuscript'.

**REPLY: Included**

If you would like to make changes to your financial disclosure, please include your updated statement in your cover letter. Guidelines for resubmitting your figure files are available below the reviewer comments at the end of this letter.

We look forward to receiving your revised manuscript.

Kind regards,

Jane Elizabeth Aspell, PhD
Academic Editor
PLOS ONE

Journal Requirements:

When submitting your revision, we need you to address these additional requirements.

1. Please ensure that your manuscript meets PLOS ONE's style requirements, including those for file naming. The PLOS ONE style templates can be found at
<https://journals.plos.org/plosone/s/file?id=wjVg/PLOSOne_formatting_sample_main_body.pdf> and
<https://journals.plos.org/plosone/s/file?id=ba62/PLOSOne_formatting_sample_title_authors_affiliations.pdf>

**REPLY: Having read through the style templates we can confirm we have completed the formatting correctly. Our citations previously used brackets () but we have since changed this to []. We have also ensured all tables are presented directly underneath the paragraph they were referenced in. We have also removed the URL of the DOIs in our references, to present them as doi: DOI/xxxx.**

2. We note that the grant information you provided in the ‘Funding Information’ and ‘Financial Disclosure’ sections do not match.
When you resubmit, please ensure that you provide the correct grant numbers for the awards you received for your study in the ‘Funding Information’ section.

**REPLY: Resolved**

3. Please review your reference list to ensure that it is complete and correct. If you have cited papers that have been retracted, please include the rationale for doing so in the manuscript text, or remove these references and replace them with relevant current references. Any changes to the reference list should be mentioned in the rebuttal letter that accompanies your revised manuscript. If you need to cite a retracted article, indicate the article’s retracted status in the References list and also include a citation and full reference for the retraction notice.

**REPLY: We can confirm that the reference list has been thoroughly checked, and correctly listed. To improve the manuscript according to the suggestions of the reviewers, we have added several additional references which are visible in tracked changes both in-text and in the bibliography. For clarity we state them here (with their corresponding numbers for ease to cross reference in text):**

20.	Zamariola G, Frost N, Van Oost A, Corneille O, Luminet O. Relationship between interoception and emotion regulation: New evidence from mixed methods. J Affect Disord. 2019;246:480–5. doi: 10.1016/J.JAD.2018.12.101

21.	Desdentado L, Miragall M, Llorens R, María Baños R. Disentangling the role of interoceptive sensibility in alexithymia, emotion dysregulation, and depression in healthy individuals. Current Psychology. 2023;42:20570–82. do: 10.1007/s12144-022-03153-4

33.	Bornstein MH, Putnick DL, Rigo P, Esposito G, Swain JE, Suwalsky JTD, et al. Neurobiology of culturally common maternal responses to infant cry. Proc Natl Acad Sci U S A. 2017;114(45):E9465–73. doi: 10.1073/pnas.1712022114

52.	MacCormack JK, Castro VL, Halberstadt AG, Rogers ML. Mothers’ interoceptive knowledge predicts children’s emotion regulation and social skills in middle childhood. Social Development. 2020;29(2):578–99. doi: 10.1111/sode.12418

64.	Gallagher S, Cole J. Body Image and Body Schema in a Deafferented Subject. The Journal of Mind and Behavior. 1995;16(4):369–90.

65.	Eshkevari E, Rieger E, Longo MR, Haggard P, Treasure J. Persistent body image disturbance following recovery from eating disorders. International Journal of Eating Disorders. 2014;47(4):400–9. doi: 10.1002/EAT.22219

112.	Christie H, Hiscox L V., Halligan SL, Creswell C. Examining harmful impacts of the COVID-19 pandemic and school closures on parents and carers in the United Kingdom: A rapid review. JCPP Advances. 2022;2(3):e12095. doi: 10.1002/jcv2.12095

113.	Xue B, McMunn A. Gender differences in unpaid care work and psychological distress in the UK Covid-19 lockdown. PLoS One. 2021;16(3):e0247959. doi: 10.1371/journal.pone.0247959

114.	Feldman R, Magori-Cohen R, Galili G, Singer M, Louzoun Y. Mother and infant coordinate heart rhythms through episodes of interaction synchrony. Infant Behav Dev. 2011;34(4):569–77. doi: 10.1016/j.infbeh.2011.06.008
